# Sciatic nerve fractional anisotropy and neurofilament light chain protein are related to sensorimotor deficit of the upper and lower limbs in patients with type 2 diabetes

**DOI:** 10.3389/fendo.2023.1046690

**Published:** 2023-03-14

**Authors:** Zoltan Kender, Johann M. E. Jende, Felix T. Kurz, Dimitrios Tsilingiris, Lukas Schimpfle, Alba Sulaj, Ekaterina von Rauchhaupt, Hannelore Bartl, Christoph Mooshage, Jens Göpfert, Peter Nawroth, Stephan Herzig, Julia Szendroedi, Martin Bendszus, Stefan Kopf

**Affiliations:** ^1^ Department of Endocrinology, Diabetology and Clinical Chemistry (Internal Medicine 1), Heidelberg University Hospital, Heidelberg, Germany; ^2^ German Center of Diabetes Research [Deutsches Zentrum für Diabetesforschung (DZD)], München, Germany; ^3^ Department of Neuroradiology, Heidelberg University Hospital, Heidelberg, Germany; ^4^ Department of Radiology, German Cancer Research Center, Heidelberg, Germany; ^5^ NMI Natural and Medical Sciences Institute at the University of Tübingen, Reutlingen, Germany; ^6^ Joint-IDC Institute for Diabetes and Cancer, Heidelberg University, Heidelberg, Germany; ^7^ Joint-IDC Institute for Diabetes and Cancer, Helmholtz-Zentrum Munich, Munich, Germany

**Keywords:** magnetic resonance neurography, diffusion tensor imaging, fractional anisotropy, diabetic neuropathy, quantitative sensory testing, neurofilament light chain protein

## Abstract

**Background:**

Diabetic sensorimotor polyneuropathy (DSPN) is one of the most prevalent and poorly understood diabetic microvascular complications. Recent studies have found that fractional anisotropy (FA), a marker for microstructural nerve integrity, is a sensitive parameter for the structural and functional nerve damage in DSPN. The aim of this study was to investigate the significance of proximal sciatic nerve’s FA on different distal nerve fiber deficits of the upper and lower limbs and its correlation with the neuroaxonal biomarker, neurofilament light chain protein (NfL).

**Materials and methods:**

Sixty-nine patients with type 2 diabetes (T2DM) and 30 healthy controls underwent detailed clinical and electrophysiological assessments, complete quantitative sensory testing (QST), and diffusion-weighted magnetic resonance neurography of the sciatic nerve. NfL was measured in the serum of healthy controls and patients with T2DM. Multivariate models were used to adjust for confounders of microvascular damage.

**Results:**

Patients with DSPN showed a 17% lower sciatic microstructural integrity compared to healthy controls (*p*<0.001). FA correlated with tibial and peroneal motor nerve conduction velocity (NCV) (r=0.6; *p*<0.001 and r=0.6; *p*<0.001) and sural sensory NCV (r=0.50; *p*<0.001). Participants with reduced sciatic nerve´s FA showed a loss of function of mechanical and thermal sensation of upper (r=0.3; p<0.01 and r=0.3; *p*<0.01) and lower (r=0.5; *p*<0.001 and r=0.3; *p*=<0.01) limbs and reduced functional performance of upper limbs (Purdue Pegboard Test for dominant hand; r=0.4; *p*<0.001). Increased levels of NfL and urinary albumin-creatinine ratio (ACR) were associated with loss of sciatic nerve´s FA (r=-0.5; *p*<0.001 and r= -0.3, *p*= 0.001). Of note, there was no correlation between sciatic FA and neuropathic symptoms or pain.

**Conclusion:**

This is the first study showing that microstructural nerve integrity is associated with damage of different nerve fiber types and a neuroaxonal biomarker in DSPN. Furthermore, these findings show that proximal nerve damage is related to distal nerve function even before clinical symptoms occur. The microstructure of the proximal sciatic nerve and is also associated with functional nerve fiber deficits of the upper and lower limbs, suggesting that diabetic neuropathy involves structural changes of peripheral nerves of upper limbs too.

## Introduction

Diabetic sensorimotor peripheral neuropathy (DSPN) affects more than half of patients with diabetes mellitus (DM) (1), resulting in increased morbidity and mortality, reduced quality of life and constitutes a considerable economic burden for health care systems (2, 3). In DSPN, structural and functional changes are observed in the whole spectrum of peripheral nervous system components including myelinated and unmyelinated nerve fibers. Even though preclinical and clinical studies have identified several underlying molecular mechanisms contributing to the pathophysiology of DSPN, the pathogenesis of DSPN remains to be elucidated (4, 5). Moreover, promising data from therapeutic applications in animal models have not yielded meaningful results in clinical trials. In addition, owing to the frequent lack of symptoms, the condition remains typically undiagnosed until later stages. Although DSPN typically predominantly affects the lower extremities, recent studies have shown that loss of sensorimotor functions of the upper limb are also frequent, but often remain underdiagnosed in patients with DM (6, 7). Contrary to the progression of clinical symptoms, previous studies on magnetic resonance neurography (MRN) showed predominantly proximally localized nerve damage in DSPN, but its relevance on different nerve fiber functions remained unclear (8–10). Validated diagnostic measures such as electrophysiological testing (EPT) and quantitative sensory testing (QST) can be used in the early detection of subclinical and asymptomatic DSPN (4, 11). These require a high degree of expertise, are time-consuming and expensive, and therefore are used mostly in the research setting. Notably, QST is considered to be a subjective method. Consequently, there is an obvious need for the establishment of objective and non-invasive methods or biomarkers that allow early detection of nerve fiber damage, to gain a better understanding of the underlying pathomechanisms and natural course of DSPN and evaluate new therapeutic options. Ideally, such methods should accurately and reproducibly detect DSPN and its progression.

Magnetic resonance neurography (MRN) can visualize and characterize peripheral nerve structures including internal fascicular patterns. Previous studies have shown that MRN is able to detect and localize peripheral nerve lesions (12, 13). Surprisingly, MRN studies revealed a proximal predominance of nerve lesions in DSPN (9). Diffusion tensor imaging (DTI) is a well-established method to measure microstructural integrity of peripheral and central nerve fibers used quantitative measures such as fractional anisotropy (FA) (14, 15). Recent studies have shown a good diagnostic accuracy of DTI for the detection of DSPN diagnosed by clinical symptoms and signs in patients with type 1 and type 2 diabetes (10, 16). In an experimental model, DTI was used to assess axonal regeneration after sciatic nerve crush (17). However, the relation of nerve fiber integrity assessed by DTI to the various nerve fiber functions of upper and lower extremities and axonal damage has not been evaluated. The prevalence of microstructural changes in patients with asymptomatic preclinical DSPN is yet unclear.

Currently, no blood-based or urine-based biomarkers for DSPN with diagnostic and predictive capacity are available for routine clinical use. Neurofilaments are neuronal proteins which levels increase in in blood and cerebrospinal fluid following axonal damage and as such, they constitute attractive candidate biomarkers in various neurodegenerative conditions (18, 19). Neurofilament light chain (NfL) is a cytoplasmatic protein with high expression in myelinated axons (20). Its role as a biomarker has been described in different neurodegenerative diseases, such as multiple sclerosis, Alzheimer disease and motoneuron diseases (19). Some preclinical and clinical studies showed an association of NfL and peripheral nerve diseases, especially in inflammatory and toxic nerve damage (21–24). One prior study showed associations between NfL and altered nerve function, especially hyperalgesia in patients with type 2 diabetes (T2DM) (25). However, the role of NfL in DSPN remained unclear.

The aim of this study was to investigate the association of extensively assessed nerve function parameters with the microstructure of the sciatic nerve as assessed from MRN (FA) and the neuroaxonal biomarker NfL in order to evaluate the role of proximal nerve damage in different nerve fiber functions and to better understand the pathophysiological background of nerve damage in DSPN. We hypothesized that the FA of sciatic nerve is associated with (i) a marker of axonal damage and (ii) loss of different nerve fiber functions of the upper and lower limb, (iii) even in patients with preclinical symptomatic DSPN.

## Methods

### Study design and participants

Participants were assessed from outpatient facilities of Heidelberg University Hospital between June 2017 and August 2020. Patients with diabetes were diagnosed according to the guidelines of the German Diabetes Association (26). Healthy control subjects had to perform an oral glucose tolerance test. Overall exclusion criteria were age <18, type 1 diabetes, pregnancy, and any contraindications for MR imaging. Individuals with known neurological diseases or neuropathy-associated risk factors such as Parkinson’s disease, multiple sclerosis, lumbar surgery or disc extrusion, entrapment syndromes, alcohol abuse, hypovitaminosis, malignant or infectious diseases were excluded. All participants gave written informed consent. The study was is conducted according to the Declaration of Helsinki (2013), approved by the local ethics committee of the University of Heidelberg (local ethics number S-383/2016, clinicaltrials.gov identifier NCT03022721).

### Clinical and electrophysiological examination

All participants underwent assessment of detailed medical history and anthropometric measurements. Blood was drawn and urine was taken under fasting conditions in the morning to measure HbA1c, lipid profile, albumin/creatinine ratio, and glomerular filtration rate (GFR). To estimate GFR the Chronic Kidney Disease Epidemiology Collaboration (CKD-EPI) formula was used (27).

All participants underwent a routine clinical examination including the neuropathy deficit score (NDS) and neuropathy symptom score (NSS) (11, 28).

The fine motor functions of the hands and manual dexterity were tested using a Purdue Pegboard Test (Model 32020; Lafayette Instrument Co., Lafayette, Indiana USA), as previously described (6, 29).

The EPT using the Viking IV electromyography system (Nicolet, Middleton, Wisconsin, USA) of the right leg includes distal motor latencies of the tibial and peroneal nerve, compound motor and sensory action potentials (CMAPs and SNAPs, respectively) and nerve conduction velocities (NCV) of the tibial, peroneal and sural nerves; all tests were done by the same trained technician (30). Participants without recordable sural NCV and SNAP were assigned the lowest measured NVC, and a SNAP of 0. Additionally, EPT of the sensory and motor component on the median and ulnar nerves were performed in 6 control subjects and 38 patients with type 2 diabetes (31). Skin temperature was at least 32°C throughout the examination.

We defined DSPN according to following criteria:1) a score of ≥ 3 in NSS or NDS (a higher score was chosen); 2) Abnormal results of nerve conduction studies in at least two different nerves (28, 32, 33).

### Quantitative sensory testing

QST was performed on one foot and one hand according to the protocol of the German Research Network on Neuropathic Pain (DFNS), as previously published (34). Complete QST can determine neuropathic deficits and hyperalgesia with the following parameters: cold detection threshold (CDT); warm detection threshold (WDT); thermal sensory limen (TSL); cold pain threshold (CPT); heat pain threshold (HPT); pressure pain threshold (PPT); mechanical pain threshold (MPT); mechanical pain sensitivity (MPS); wind-up ratio (WUR); mechanical detection threshold (MDT); vibration detection threshold (VDT); dynamic mechanical allodynia (DMA); and paradoxical heat sensation (PHS) (11, 34). CDT, WDT, and TSL reflect thermal detection of small fibers (Aδ and C fibers), while CPT, HPT, PPT, and particularly MPS, and MPT represent nociceptive sensation of small fibers (Aδ and C fibers). MDT and VDT reflect tactile sensation (mechanical detection) of larger Aβ fibers (12).

### MRN imaging protocol

MRN examinations were performed of the right tight using a 3-Tesla MR-scanner (Magnetom TIM-TRIO, Siemens Healthcare, Erlangen, Germany) with a 15-channel transmit-receive extremity coil in all participants, as previously described (8, 33). MRN sequences were centered in all participants on the sciatic nerve’s bifurcation to ensure that the anatomic region visualized by MRN was comparable in all participants. MRIs were acquired from the following sequences:

1) Axial high resolution T2-weighted turbo spin echo (TSE) 2D sequence with spectral fat saturation, and the following parameters: relaxation time (TR) = 5970 ms, echo time (TE) = 55ms, field of view (FOV) = 160 x 160 mm^2^, matrix size = 512 x 512, slice thickness = 4 mm, interslice gap = 0.79mm, voxel size = 0.3 x 0.3 x 4.0 mm^3^, 24 slices, 24 acquired images.
2) Axial fat-suppressed, diffusion-weighted 2-dimensional echo-planar sequence with the following parameters: TR = 5100 ms; TE=92.8 ms; b = 0 and 1000 s/mm^2^; directions = 20; field of view 160 x 160 mm^2^; matrix size 128 x 128; slice thickness 4 mm; voxel size 1.3 x 1.3 x 4 mm^3^; no interslice gap, 3 averages, 24 slices, 1512 acquired images.

### Image post-processing and statistical analysis

Nordic BRAINEX software, a Food and Drug Administration (FDA) approved processing software was used to process and analyze images with automated calculation and reconstruction of nerve fiber tracts in DTI (35, 36).. We followed the DTI module instructions for Nordic BRANEX (Nordic Neuro Lab, Bergen, Norway). T2-weighted and diffusion weighted sequences were analyzed by two trained neuroradiologists with 7 and 8 years of experience in MRN imaging, respectively. Both were blinded to clinical and demographic patient data. Based on previous studies on DTI of the sciatic nerve, the nerve was automatically segmented with a threshold of > 0.1 for the nerve’s FA. The average FA was calculated automatically (36).

### Quantification of neurofilament light chain protein

Serum blood samples were collected from all participants and centrifuged within 1 hour and stored at −80°C. NfL was measured within 4 years after collection of serum samples. For analysis of the concentration of NfL in serum samples a Simoa immunoassay (Quanterix, Billerica, MA, USA) was used, as previously described (25). For this study samples were analyzed in two independent assay runs on two different days using the same kit lots on a Simoa HD-X analyzer (Quanterix). Sample concentrations were calculated based on individual standard curves for each assay run using the Simoa HD-X Software (Version 3.1.2011.30002, Quanterix). The quality control (QC) samples provided by the kit manufacturer were included in both assay-runs and met the acceptance range specified with the kit. The inter-run coefficient of variation (CV) of the two levels of QCs was 6.4 and 11.9%.

### Statistical analysis

Data sets of continuous variables were tested for normal distribution using the Kolmogorov-Smirnov test. Descriptive statistics are expressed as absolute numbers and percentages (n, %), means ± standard deviations or medians [25, 75 interquartile range] for categorical, normally, and non-normally distributed continuous data, respectively. One-way-ANOVA or the Kruskal-Wallis non-parametric test were used for comparisons across the three subgroups regarding normally and non-normally distributed continuous variables, respectively. In case of significant differences, *post-hoc* pairwise comparisons with Bonferroni correction were applied. was used to compare means of normally distributed variables, A chi-squared test was applied to compare categorical data. The Pearson correlation coefficient was used to examine the association of DTI (dependent variable) with factors pertinent to peripheral nerve function (independent variable). In order to ascertain the independence of observed correlations, multivariable linear regression models were used with inclusion of multiple potential confounders as covariates. The normality of regression residuals was tested through the Kolmogorov-Smirnov test and residual Q-Q plots and histograms was deemed adequate. For all tests the level of significance was defined at p<0.05. SPSS Version 23.0 (IBM-SPSS, Chicago, IL, USA) was used for statistics, and GraphPad Prism 7 for illustrating figures (GraphPad Software Inc. La Jolla, CA; USA).

## Results

### Participants characteristics and MRN imaging

The study cohort consisted of 99 participants, 30 with normal glucose tolerance, 40 with T2DM without DSPN and 29 with T2DM with DSPN. There were no differences in sex distribution, BMI, eGFR, and high-sensitive C-reactive protein levels. Patients with T2DM and DSPN were slightly older than healthy controls. Participants with T2DM were slightly older than controls (62.9 *vs*. 57.5 years, p=0.010), chiefly due to the difference between those with T2DM and DSPN versus healthy controls (63.7 *vs*. 57.5 years, p=0.038 in *post-hoc* comparison). There were no differences in age, sex, diabetes duration, statin use and established cardiovascular disease (CVD) between the subgroups of participants with T2DM with and without DSPN. As expected, participants with T2DM showed higher fasting plasma glucose, HbA1c and triglycerides compared to control subjects. Neuropathy scores were found to be highest among those with T2DM and DSPN compared to the other two groups and comparable between glucose tolerant controls and T2DM patients without DSPN (**Table 1**).

**Table 1:** Clinical characteristics of study participants.

Parameter	Control group (n = 30)	w/o DSPN (n = 40)	with DSPN (n = 29)
Age [years]	57.5 ± 10.9	62.3 ± 8.7	63.7 ± 8.9^#^
Sex [female]	18 (60.0)	15 (37.5)	9 (31.0)
Duration of diabetes [years]	–	7.0 [4.0, 15.0]	8.5 [3.8, 16.3]
Statin use [yes]	5 (16.7)	18 (45.0)^*^	16 (55.2)^#^
Known CVD [yes]	0 (0.0)	4 (10.0)	8 (27.6)^#^
BMI [kg/m^2^]	27.9 ± 6.1	29.1 ± 4.9	30.7 ± 4.3
Fasting glucose [mg/dL]	94.1 ± 8.3	139.2 ± 38.4^*^	145.6 ± 36.0^#^
HbA1c [%]	5.5 ± 0.4	6.9 ± 1.1^*^	7.0 ± 1.1^#^
HbA1c [mmol/mol]	36.8 ± 4.6	51.7 ± 12.5^*^	53.1 ± 11.8^#^
eGFR [mL/min/1.73m^2^]	92.1 ± 13.0	92.12 ± 16.8	88.2 ± 16.2
hsCRP [mg/L]	0.70 [0.40, 1.58]	1.70 [0.58, 3.37]	1.26 [0.52, 3.30]
Cholesterol [mg/dL]	211.3 ± 38.9	189.9 ± 41.8	193.6 ± 40.8
Albumin/Creatinine Ratio (mg/g)	5.7 [4.1, 8.3]	8.6 [4.0, 17.4]	9.8 [5.7, 35.4] ^#^
Neuropathy symptom score [/10]	0.0 [0.0, 0.0]	0.0 [0.0, 5.0]	6.5 [6.0, 8.0] ^#§^
Neuropathy deficit score [/10]	0.0 [0.0, 1.0]	2.0 [0.0, 2.0]	6.0 [4.0, 8.0] ^#§^
Fractional anisotropy	0.48 ± 0.06	0.45 ± 0.06	0.40 ± 0.07^#§^
NfL [pg/ml]	8.96 ± 3.64	12.24 ± 5.93	13.93 ± 7.30^#^

Data are n (%), means ± SD or medians [25, 75 interquartile range]. ^*^p < 0.05 controls vs. patients without DSPN; ^#^p < 0.05 controls vs. patients with DSPN; ^§^p < 0.05 without DSPN vs. with DSPN.

BMI, body mass index; hsCRP, high-sensitivity C-reactive protein; CVD, cardiovascular disease; eGFR, estimated glomerular filtration rate; HbA1c, Hemoglobin A1c.

**Figure 1:**
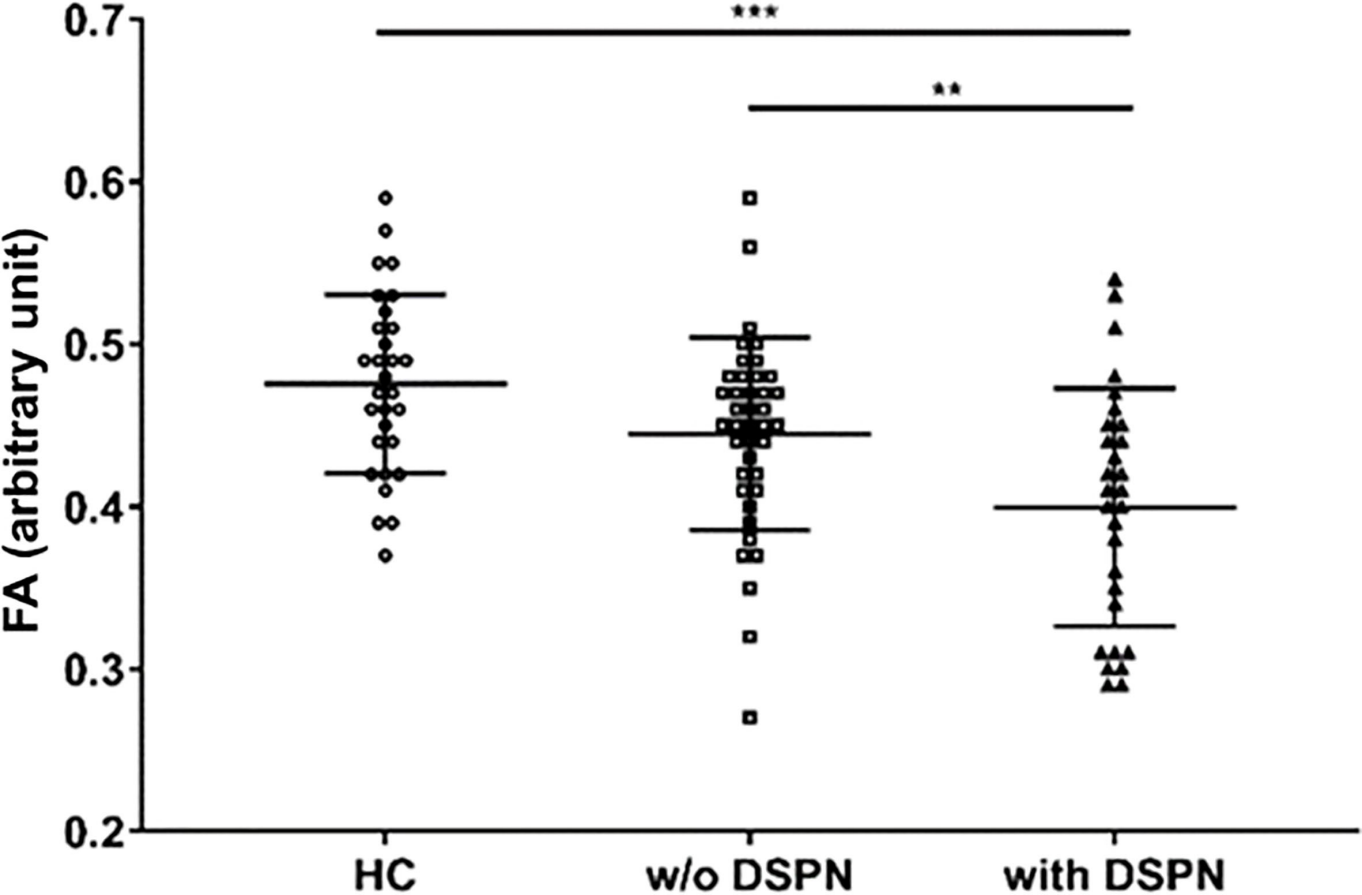
Quantification of sciatic nerve’s fractional anisotropy in control participants (n=30), participants with type 2 diabetes without DSPN (n=40), and participants with type 2 diabetes and DSPN (n=29). FA, fractional anisotropy; HC, healthy control; DSPN, Diabetic sensorimotor polyneuropathy. **p<0.01; ***p<0.001.

The mean FA values were lower among those with T2DM and DSPN compared with the other two groups (0.40 *vs*. 0.48 and 0.45 for T2DM with DSPN *vs*. controls and T2DM without DSPN, respectively, p-ANOVA < 0.001 **Figure 1**). Mean NfL concentration gradually increased across healthy, T2DM without and with DSPN (8.96 *vs*. 12.24 *vs*. 13.93, respectively p-ANOVA= 0.008).

### Association of MRN imaging with neuropathic deficits of the lower limb

As a next step, the associations between the FA of the sciatic nerve and neuropathic deficits of the lower limbs were investigated. In total, the findings were suggestive of a robust association of FA values to an impaired peripheral sensory and motor nerve status (**Table 2**). In univariable analysis, FA showed negative correlations with clinical neuropathy scores (NSS and NDS, r= -0.517, *p*<0.001 and r=-0.034, *p*=0.001, respectively) as well as tibial and peroneal distal motor latencies, and positive with tibial and peroneal motor NCVs and amplitudes, as well as sural sensory NCVs and amplitudes(p<0.001 for all parameters). With the exception of neuropathy symptom score, these findings remained essentially unchanged in two multivariable regression models adjusted for various parameters which may have confounded the observed associations (model 1 adjusted for age, sex, body mass index (BMI) and waist–hip ratio (WHR), model 2 additionally for HbA1c, eGFR, total cholesterol, statin use and manifest CVD). Of note, no significant effects of HbA1c on the tested values were noted in the adjusted models (data not shown).

**Table 2:** Correlations of sciatic nerve´s FA (dependent variable) with appropriate clinical parameters and nerve conduction studies of the lower limb participants (independent variables) for all study participants.

	*Univariable*		*Model 1*		*Model 2*	
Parameter	r	p	Beta	P	Beta	p
Neuropathy symptom score [/10]	-0.517	**<0.001**	-0.195	0.061	-0.180	0.104
Neuropathy deficit score [/10]	-0.341	**0.001**	-0.388	**<0.001**	-0.390	**<0.001**
Tibial nerve motor NCV [m/s]	0.561	**<0.001**	0.520	**<0.001**	0.509	**<0.001**
Tibial CMAP [mV]	0.599	**<0.001**	0.488	**<0.001**	0.496	**<0.001**
Tibial distal motor latency [ms]	-0.516	**<0.001**	-0.433	**<0.001**	-0.464	**<0.001**
Peroneal motor NCV [m/s]	0.563	**<0.001**	0.486	**<0.001**	0.491	**<0.001**
Peroneal CMAP [mV]	0.581	**<0.001**	0.513	**<0.001**	0.527	**<0.001**
Peroneal distal motor latency [ms]	-0.514	**<0.001**	-0.432	**<0.001**	-0.463	**<0.001**
Sural sensory NCV [m/s] ^†^	0.496	**<0.001**	0.357	**0.001**	0.403	**0.001**
Sural SNAP [µV] ^†^	0.512	**<0.001**	0.451	**<0.001**	0.455	**<0.001**

Data are means ± SD. ^†^n=98.

Beta: standardized regression coefficient, CMAP, compound motor action potential; NCV, nerve conduction velocity; SNAP, sensory nerve action potential.

Model 1: Adjusted for age, sex, BMI, waist-to-hip ratio.

Model 2: Additionally, adjusted for HbA1c, eGFR, Total Cholesterol, Statin use and manifest CVD.

Bold values are stat. significant values (significance was defined at p<0.05).

### Association of MRN imaging with neuropathic deficits of the upper limb

**Table 3** shows the associations of sciatic nerves’ FA with neuropathic deficits of the upper limb. Higher FA values were independently associated with increased sensory and motor NCV of the median (r= 0.545, *p*<0.001 and r=0.434, *p*=0.004) and ulnar nerves (r=0.431, *p*=0.003 and r=0.541, *p*<0.001) as well as CMAP of the ulnar nerve (r= 0.452, p=0.002) and SNAP of the median and ulnar nerves (r=0.516 and r= 0.522, *p*<0.001). Higher FA values were associated with higher fine motor dexterity in all four domains of the Purdue Pegboard Test in univariable analysis. In the adjusted models however, this correlation remained significant for the dominant hand only (*p*=0.01).

**Table 3:** Correlations of sciatic nerve´s FA (dependent variable) with nerve conduction studies and functional parameters of the upper limb participants (independent variables) for all study participants.

	*Univariable*		*Model 1*		*Model 2*	
Parameter	*r*	*P*	*Beta*	*P*	*Beta*	*p*
Median motor NCV [m/s] ^†^	0.545	**<0.001**	0.580	**<0.001**	0.536	**<0.001**
Median CMAP [mV] ^†^	0.277	0.165	0.243	0.167	0.055	0.786
Ulnar motor NCV [m/s] ^†^	0.431	**0.003**	0.436	**0.011**	0.395	**0.027**
Ulnar CMAP [mV] ^†^	0.452	**0.002**	0.385	**0.018**	0.403	**0.017**
Median sensory NCV [m/s] ^†^	0.434	**0.004**	0.534	**0.003**	0.502	**0.006**
Median SNAP [µV] ^†^	0.516	**<0.001**	0.437	**0.018**	0.481	**0.007**
Ulnar sensory NCV [m/s] ^†^	0.541	**<0.001**	0.551	**0.001**	0.491	**0.003**
Ulnar SNAP [µV] ^†^	0.522	**<0.001**	0.491	**0.010**	0.659	**<0.001**
Pegboard dominant hand [/30s] ^‡^	0.425	**<0.001**	0.300	**0.010**	0.320	**0.011**
Pegboard non-dominant hand [/30s] ^‡^	0.414	**<0.001**	0.230	0.066	0.221	0.097
Pegboard both hands [/30s] ^‡^	0.390	**<0.001**	0.222	0.056	0.233	0.066
Pegboard Assembly Test [/60s] ^§^	0.361	**0.001**	0.109	0.421	0.179	0.244

Data are means ± SD.

^†^n=44.

^‡^score equals number of pegs placed in 30 sec.

^§^score equals number of pegs, collars, and washers assembled in 60 min.

CMAP, compound motor action potential; NCV, nerve conduction velocity; SNAP, sensory nerve action potential.

Bold values are stat. significant values (significance was defined at p<0.05).

### Association of MRN imaging with quantitative sensory testing

In the lower extremity, FA exhibited positive and independent associations most prominently with QST domains indicative of medium (mechanical pain threshold and mechanical pain sensitivity) and large (mechanical detection threshold and vibration detection threshold) fiber functional status. Regarding small fiber indices, a significant and independent association was noted with the cold pain threshold (r=0.301, *p*=0.004 in univariable analysis) (**Table 4A**). After grouping individual QST domains into compound scores on a functional basis, FA was shown to positively correlate with the compound z-scores of thermal and mechanical detections (r=0.26, *p*=0.008 and r=0.536, *p*<0.001) as well mechanical pain (r=0.379, *p*=0.001) on the feet measured by quantitative sensory testing (QST). In multivariable regression analysis, the associations with mechanical detection and pain remained constant, while the association with thermal detection was marginally non-significant (**Table 4A**).

**Table 4:** Correlations of sciatic nerve´s FA (dependent variable) with z-scores of QST parameters and compound z-scores of the feet and hands in all participants (independent variables).

Foot							
(A)	Univariable		*Model 1*		*Model 2*		
	R	*p*	*beta*	*p*	*Beta*	*P*	
zCDT	**0.306**	**0.003**	0.190	0.059	0.190	0.064	***small fibres***
zWDT	**0.216**	**0.038**	0.182	0.108	0.180	0.124	
zTSL	0.183	0.079	0.178	0.065	0.182	0.062	
zCPT	**0.301**	**0.004**	**0.237**	**0.015**	**0.277**	**0.007**	
zHPT	-0.023	0.826	-0.017	0.859	-0.018	0.862	
zPPT	0.058	0.581	0.053	0.584	0.071	0.475	**medium fibres**
zMPT	**0.352**	**0.001**	**0.325**	**0.001**	**0.353**	**<0.001**	
zMPS	**0.216**	**0.038**	**0.212**	**0.027**	**0.200**	**0.044**	
zWUR	-0.042	0.694	-0.060	0.546	-0.051	0.623	**large fibres**
zMDT	**0.409**	**<0.001**	**0.311**	**0.002**	**0.310**	**0.002**	
zVDT	**0.444**	**<0.001**	**0.311**	**0.003**	**0.310**	**0.006**	
Thermal detection	**0.266**	**0.008**	0.190	0.054	0.191	0.057	**Compound scores**
Mechanical detection	**0.536**	**<0.001**	**0.374**	**<0.001**	**0.386**	**<0.001**	
Thermal pain	0.150	0.176	0.111	0.258	0.131	0.202	
Mechanical pain	**0.379**	**0.001**	**0.316**	**0.001**	**0.338**	**0.001**	
*Hand*							
(B)	Univariable		*Model 1*		*Model 2*		
	R	*p*	*beta*	*p*	*beta*	*P*	
zCDT	**0.192**	**0.065**	0.164	0.090	0.168	0.093	***small fibres***
zWDT	**0.290**	**0.005**	**0.236**	**0.015**	**0.258**	**0.009**	
zTSL	**0.317**	**0.002**	**0.271**	**0.006**	**0.300**	**0.003**	
zCPT	-0.079	0.452	0.065	0.531	0.060	0.568	
zHPT	0.149	0.154	0.186	0.059	**0.199**	**0.047**	
zPPT	-0.107	0.308	-0.107	0.287	-0.098	0.348	**medium fibres**
zMPT	-0.115	0.271	-0.013	0.897	-0.008	0.939	
zMPS	-0.101	0.335	-0.003	0.975	-0.007	0.942	
zWUR	-0.108	0.316	-0.161	0.110	-0.184	0.087	**large fibres**
zMDT	**0.253**	**0.015**	**0.189**	**0.050**	**0.237**	**0.017**	
zVDT	0.197	0.058	**0.192**	**0.049**	**0.203**	**0.047**	
Thermal detection	**0.291**	**0.004**	**0.247**	**0.011**	**0.263**	**0.008**	**Compound scores**
Mechanical detection	**0.266**	**0.008**	**0.227**	**0.018**	**0.264**	**0.008**	
Thermal pain	0.047	0.654	0.152	0.131	0.157	0.124	
Mechanical pain	-0.126	0.138	-0.055	0.595	-0.50	0.639	

QST parameters are categorized by fiber size.

Bold values are stat. significant values (significance was defined at p<0.05).

Regarding the hand sciatic FA showed positive associations with small (warm detection threshold and thermal sensory limen) and large fiber functions (mechanical detection threshold and vibration detection threshold). Likewise, there were independent positive correlations between FA and compound z-scores of thermal and mechanical detections of the hand (r=0.291, *p*=0.004 and r=0.266, *p*=0.008) (**Table 4B**).

### Association of MRN imaging with clinical and serological biomarkers

**Figure 2:**
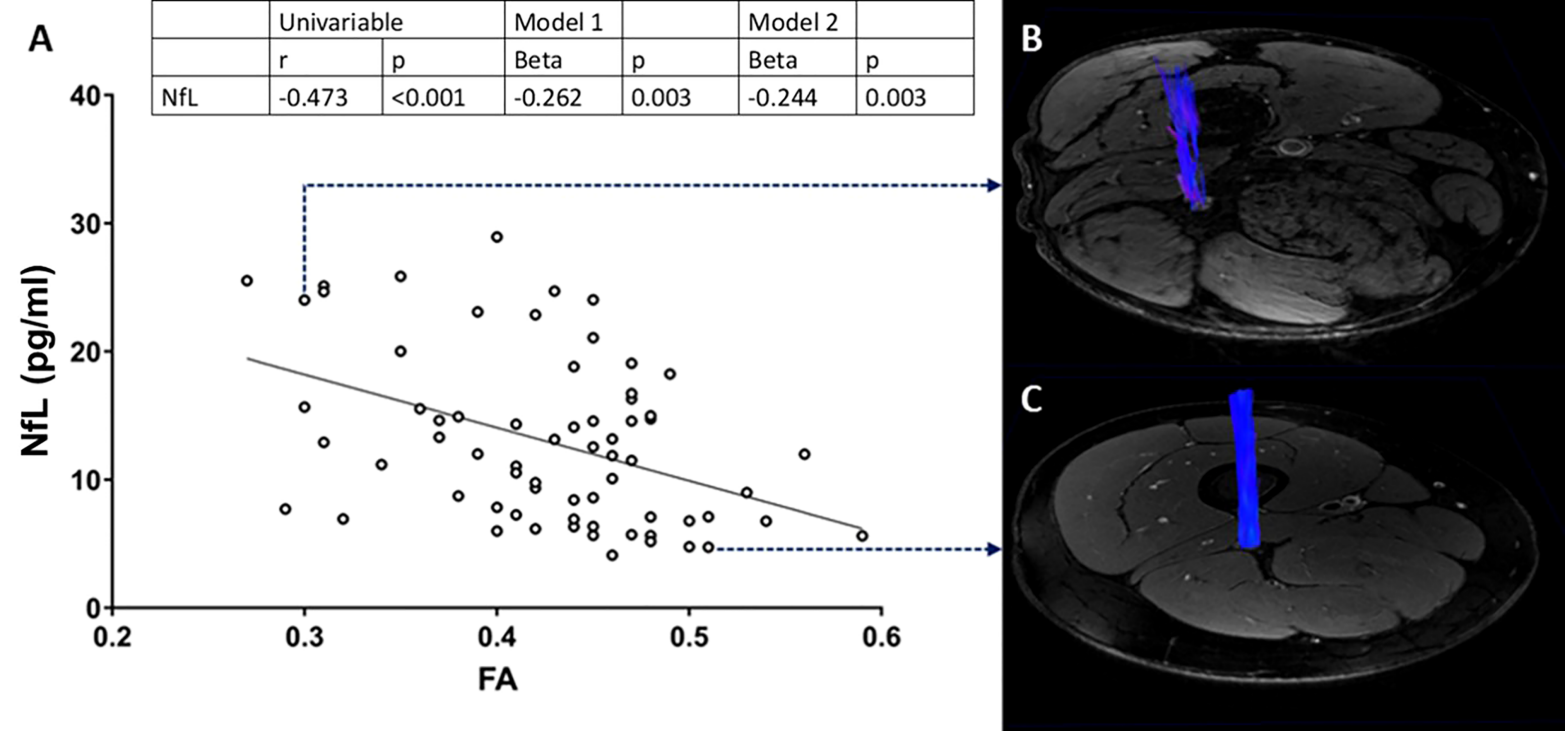
Correlation analysis of FA on the sciatic nerve with plasma NfL **(A)**. Reconstructed, 3D fiber track of the right sciatic nerve of a patient with decreased FA and increased NfL **(B)**, and a heathy control **(C)**. FA, fractional anisotropy; NfL, neurofilament light chain protein.

In order to test the hypothesis that sciatic nerve FA values may reflect peripheral sensory axonal damage in the lower extremity, we investigated the association between FA and circulating levels of axonal biomarker NfL, using a similar modelling approach as above. There was a negative correlation between the two parameters, which remained unaffected in multivariable analysis (r=-0.244, p=0.003) (**Figure 2A**). Representative sciatic nerve fiber tracts from an individual with decreased FA and increased NfL and from a healthy individual with normal FA and NfL are depicted in **Figures 2B, C**.

As an additional step, the relationship between FA and albumin-to-creatinine ratio (ACR), a surrogate of microvascular damage, was examined. Due to its extremely skewed distribution the log-transformed ACR values were used in the analysis. There was a negative correlation between FA and log-transformed ACR (r=-0.347, p= 0.001), which remained unaffected in multivariable analysis (beta=-0.251, p=0.011, beta=-0.250 p=0.015 in models 1 and 2, respectively).

When both NfL and logACR, NDS and NSS were included as independent variables in a fully adjusted multivariable model (as in model 2 previously), NDS (beta= -0.300, p=0.018) and NfL (beta= -0.390, p=0.014) but not ACR were independent predictors of FA. The effects of other included variables were non-significant (**Table 5**).

**Table 5:** Linear regression analysis for FA (dependent variable) of the sciatic nerve in all participants.

Independent variables	Beta	*p* value
Neuropathy Disability Score [/10]	-0.300	**0.018**
Neuropathy Symptom Score [/10]	-0.083	0.483
NfL [pg/ml]	-0.390	**0.014**
Log(ACR) (mg/g)	-0.052	0.634

Beta: standardized regression coefficientAnalysis is adjusted for age, gender, BMI, waist-to-hip ratio, HbA1c, eGFR, Total Cholesterol, Statin use and manifest CVD.

Bold values are stat. significant values (significance was defined at p<0.05).

## Discussion

The results of this cross-sectional study in well characterized patients with T2DM showed associations between sciatic nerve integrity as assessed from FA measured by MRN based DTI and peripheral sensory as well as motor nerve status of upper and lower limbs. To our knowledge, this study is the first to demonstrate that sciatic nerve´s FA reflects sensory loss of small, medium, and large fibers even in early stages of DSPN. Since MRN itself cannot differentiate between different nerve fiber types, the ascertained associations with alterations in different fiber functions aid to a better understanding of peripheral nerve’s FA. In line with the initial hypothesis, we found an association between reduced nerve integrity and higher serum NfL, a biomarker of axonal damage. The observed associations were independent of age, sex and other covariables, and comprised asymptomatic patients with preclinical DSPN, strengthening the notion that changes in sciatic nerve´s microstructure play an important role in the development of DSPN.

Previous studies on magnetic resonance neurography in DSPN have focused on standard imaging protocols based on morphological sequences or T2-weighted sequences (9, 37). However, with a lack of microstructural specificity. DTI is a functional magnetic resonance imaging (MRI) technique, which can visualize microstructural changes in the central and peripheral nerve system at the anatomical level of nerve fascicles (38–40). FA reflects the directional orientation of water molecules within the nerve tissue and the anisotropy of peripheral nerves (40). Recent studies have demonstrated a reduction in FA for the sciatic and tibial nerves in patients with clinically overt DSPN (10, 16, 33, 41). These studies showed an association between FA and large and medium myelinated fiber dysfunction evident by nerve conduction studies (NCS). However, these studies have not detected small fiber functions. The most sensitive clinical method for the detection and characterization of peripheral nerve fiber functional deficits is complete quantitative sensory testing (QST), which includes the estimation of thermal and mechanical detection and pain thresholds (11, 34, 42). QST has not yet been used to evaluate the clinical relevance of FA. A recent study showed that T2-weighted lesions in the sciatic nerve may be associated with a decline in medium and large fiber functions in DSPN (12). To our knowledge, our study is the first to show that DTI can detect more widespread nerve fascicle lesions, including those affecting unmyelinated medium and small fibers, as evidenced by changes in individual sensation thresholds (**Table 4**). This is of clinical importance, since route tests like neuropathy scores and NCS cannot detect unmyelinated fiber functions which serve clinically relevant functions such as pain and the regulation of microvascular blood flow. In the current study we unraveled positive and independent associations between FA and mechanical detection and pain thresholds of the foot and thermal and mechanical detection thresholds of the hands. Notably, in multivariable analysis FA showed no association with neuropathic symptoms assessed by NSS. This finding is very important since it may demonstrate that proximal nerve damage is related to distal nerve function even before clinical symptoms appear.

The pathophysiological background of the lesions detected by MRN is yet unclear. Notably, electrophysiological testing showed strong associations of sciatic nerve´s FA with NCV, an indicator of myelin damage, and even more robust with SNAP, an indicator of axonal loss. While fiber loss is considered more prominent than myelin damage in DSPN (43), both types of nerve fiber damage have previously been described in DSPN (44). In models of injury of the centrals nervous system, decreased FA is associated with the degree of axon degeneration (45–47). Axonal injury is a severe complication of DSPN and can lead to loss of sensory and motor function of peripheral nerves. An independent association of sciatic nerve´s FA with serum NfL, a known biomarker of axonal damage, was also noted. These findings support the concept that structural nerve changes in DSPN visualized with DTI are causally related to injured microstructural integrity in DSPN caused in part by axonal damage (16, 48).

Furthermore, FA showed an independent negative association with ACR, a surrogate of microvascular disease in DM, but not with glycemia as indexed by HbA1c. Several studies have shown limited effect of glycemia on the course of DSPN, indicating that other risk factors such as microvascular damage may contribute to reduced nerve tissue integrity due to the degeneration of axons and Schwann cells in DSPN (49). A previous study on QST also showed an association between DSPN and albuminuria (11). However, this correlation in current study is modest, and in a fully adjusted multivariable model including NfL and NDS, ACR did not remain an independent predictor of nerve integrity. This supports the hypothesis that changes of FA might be more specific to the restriction of intra-axonal diffusion caused by injured microstructure and axonal damage, rather than being confounded by concomitant perturbations of systemic microvascular damage in DSPN.

To our knowledge this is the first study demonstrating that sciatic nerve integrity reflects a widespread sensorimotor loss including small fiber functions not only of the lower, but also of upper extremities. This may harbor implications regarding the natural course of nerve damage in DSPN. The finding that proximal microstructural damage of the sciatic nerve shows independent associations with indices of peripheral nerve fiber damage also of the upper limb suggests that in DSPN may be viewed as a systemic condition affecting the entire peripheral nervous system. This is in line with results from a previous study showing a substantial prevalence of upper limb neuropathy in patients with T2DM (6). A further implication would include FA of the sciatic nerve as a surrogate of peripheral nervous system status as a whole, in the frame of early preclinical stages of DSPN.

Our study is limited by its cross-sectional design, which precludes any definite conclusions on the predictive value of the sciatic nerve’s FA to be drawn. However, FA also related to nerve function deficits in asymptomatic patients with DSPN, suggesting a role of microstructural changes of proximal nerves in the pathophysiology of diabetic neuropathy. Another limitation is that only a subset of MRN patients underwent NCS of the upper limb. However, to date no data is available on the relation of sciatic FA with upper limbs nerve function in patients with and without DSPN. Furthermore, DTI metrics might be influenced by the technical setup, sequence specifications and the accuracy of the software. To date, differences between DTI techniques have been assessed only in few studies which showed comparable DTI measurements across different MR imaging platforms (50, 51). It should be also noted, that systematic errors during the automated reconstruction process may impact the FA values, however the algorithm used by BRAINEX is a widely used for DTI reconstruction (52). The present study has several strengths. We included a relatively high number of well characterized patients with and without DSPN. These subgroups are very similar regarding demographic and clinical profiles. We used not only electrophysiological testing, but a complete QST to assess all nerve fiber functions, including small fiber functions and hypersensitivity. The statistical analysis was adjusted carefully for several covariates in different models.

In summary, this study demonstrates that the sciatic nerve´s microstructure is related to Nfl, a marker of axonal damage and impaired function of different nerve fiber types in DSPN. In addition, these findings show that proximal nerve damage is related to distal nerve function even before clinical symptoms occur. The sciatic nerve´s microstructure is also associated with functional deficits of the upper and lower limbs, suggesting that upper and lower limbs are simultaneously affected by nerve damage in DSPN. Further longitudinal studies are needed to detect the predictive value of the sciatic nerve’s FA for the natural course of DSPN.

## Data availability statement

The datasets presented in this article are not readily available because they are subject to national data protection laws and restrictions imposed by the ethics committee to ensure the privacy of study participants. Datasets are available from the corresponding author upon reasonable request for research purpose after anonymization and approval by the local ethics committee. Requests to access the datasets should be directed to zoltan.kender@med.uni-heidelberg.de.

## Ethics statement

The studies involving human participants were reviewed and approved by Ethikkommission der medizinischen Fakultät Heidelberg. The patients/participants provided their written informed consent to participate in this study.

## Author contributions

ZK: Conceptualization (lead); Data curation (equal); Formal analysis (lead); Investigation (equal); Methodology (equal); Visualization (lead); Project administration (equal); Writing-original draft (lead). JJ: Data curation (equal); Methodology (equal); Writing-review and editing (equal). FK: Data curation (equal); Methodology (equal); Writing-review and editing (equal). DT: Data curation (supporting); Investigation (equal); Formal analysis (equal), Writing-review and editing (equal). LS: Data curation (equal); Methodology (equal); Writing-review and editing (equal). AS: Data curation (supporting); Investigation (equal); Writing-review and editing (equal). ER: Data curation (equal); Methodology (equal); Writing-review and editing (equal). HB: Data curation (equal); Methodology (equal); Writing-review and editing (equal). CM: Data curation (equal); Methodology (equal); Writing-review and editing (equal). JG: Data curation (equal); Methodology (equal); Writing-review and editing (equal). PN: Conceptualization (equal); Data curation (equal); Formal analysis (equal); Funding acquisition (equal); Methodology (equal); Supervision (lead); Writing-review and editing (equal) SH: Data curation (supporting); Formal analysis (supporting); Writing-review and editing (equal). JS: Data curation (supporting); Formal analysis (supporting); Writing-review and editing (equal). MB: Data curation (supporting); Writing-review and editing (equal). SK: Conceptualization (equal); Data curation (equal); Formal analysis (equal); Funding acquisition (equal); Investigation (supporting); Methodology (equal); Project administration (lead); Resources (equal); Software (equal); Supervision (lead); Validation (equal); Visualization (equal); Writing-original draft (equal); Writing-review and editing (equal). All authors contributed to the article and approved the submitted version.
